# Comparative Efficacy of pHA130 Haemoadsorption Combined with Haemodialysis Versus Online Haemodiafiltration in Removing Protein-Bound and Middle-Molecular-Weight Uraemic Toxins: A Randomized Controlled Trial

**DOI:** 10.3390/toxins17080392

**Published:** 2025-08-05

**Authors:** Shaobin Yu, Huaihong Yuan, Xiaohong Xiong, Yalin Zhu, Ping Fu

**Affiliations:** Department of Nephrology, Kidney Research Institute, West China Hospital of Sichuan University, Chengdu 610041, China; yushaobinhx@wchscu.edu.cn (S.Y.); yuanhuaihong@wchscu.cn (H.Y.); xiongxiaohong@wchscu.edu.cn (X.X.); zhuyalin@wchscu.edu.cn (Y.Z.)

**Keywords:** haemoadsorption combined with haemodialysis, haemodialysis, protein-bound uraemic toxins, pHA130, indoxyl sulphate

## Abstract

Protein-bound uraemic toxins (PBUTs), such as indoxyl sulphate (IS) and p-cresyl sulphate (PCS), are poorly cleared by conventional haemodialysis (HD) or haemodiafiltration (HDF). Haemoadsorption combined with HD (HAHD) using the novel pHA130 cartridge may increase PBUT removal, and this trial aimed to compare its efficacy and safety with HDF in patients with end-stage renal disease (ESRD). In this single-centre, open-label trial, 30 maintenance HD patients were randomized (1:1:1) to HDF once every two weeks (HDF-q2w), HAHD once every two weeks (HAHD-q2w), or HAHD once weekly (HAHD-q1w) for 8 weeks, with the primary endpoint being the single-session reduction ratio (RR) of IS. The combined HAHD group (n = 20) demonstrated a significantly greater IS reduction than the HDF-q2w group (n = 10) (46.9% vs. 31.8%; *p* = 0.044) and superior PCS clearance (44.6% vs. 31.4%; *p* = 0.003). Both HAHD regimens significantly reduced predialysis IS levels at Week 8. Compared with HDF, weekly HAHD provided greater relief from pruritus and improved sleep quality, with comparable adverse events among groups. In conclusion, HAHD with the pHA130 cartridge is more effective than HDF for enhancing single-session PBUT removal and alleviating uraemic symptoms in patients with ESRD, with weekly application showing optimal symptomatic benefits.

## 1. Introduction

Chronic kidney disease (CKD) represents a significant global health burden, with the World Health Organization (WHO) reporting that CKD has emerged as one of the top ten causes of death worldwide. As CKD progresses to end-stage renal disease (ESRD), the accumulation of uraemic toxins—waste products that are normally excreted by the kidneys—poses severe multisystem risks. Uraemic toxins are generally classified by the European Uraemic Toxin Work Group (EUTox) into three categories on the basis of their biochemical properties and clearance modalities: (i) water-soluble, nonprotein-bound small molecules (e.g., urea and creatinine, typically < 500 Da) that are efficiently cleared by conventional haemodialysis (HD); (ii) middle molecules (e.g., β2-microglobulin (β2 M) and chitinase-3-like protein 1 (YKL-40), generally > 500 Da); and (iii) protein-bound uraemic toxins (PBUTs), such as indoxyl sulphate (IS) and p-cresyl sulphate (PCS), which, upon binding to proteins, exhibit a molecular weight exceeding 60,000 Da and are poorly removed by conventional dialysis techniques [1].

Recent insights into the toxicity mechanisms of uraemic toxins have underscored the deleterious effects of both PBUTs and middle to large molecules [2,3]. PBUTs, in particular, have been implicated in vascular endothelial dysfunction, smooth muscle cell proliferation, and the transdifferentiation of fibroblasts into osteoblast-like cells, leading to arterial wall thickening and calcification [4]. These pathological processes, driven by mechanisms including endothelial damage and oxidative stress, not only serve as key factors in the pathogenesis of debilitating symptoms such as uraemic pruritus and sleep disturbances but also contribute significantly to the high rates of cardiovascular and all-cause mortality observed in patients with ESRD [5,6,7,8]. Furthermore, emerging evidence indicates that YKL-40, a 40-kDa glycoprotein associated with inflammation, is closely correlated with cardiovascular events, and interventions that lower YKL-40 levels have been associated with improved overall and cardiovascular survival [9,10].

Conventional HD remains the primary modality for ESRD treatment; however, both high-flux HD (HFHD) and haemodiafiltration (HDF) have demonstrated limited efficiency in removing PBUTs and middle molecules [11,12,13]. Previous studies have reported that conventional HD or HDF results in IS and PCS reduction rates that rarely exceed 35–40%, leaving a critical unmet need for more effective removal strategies [14,15]. As a result, alternative approaches—such as extracorporeal adsorption therapies—have been proposed to increase toxin clearance [16,17].

Haemoadsorption (HA) is an extracorporeal blood purification method that uses adsorptive columns to remove toxins that are inadequately cleared by diffusion or convection [18]. The cartridges widely used in China, such as the HA130 sorbent cartridge, when integrated into the dialysis circuit prior to the haemodialyzer (haemoadsorption combined with haemodialysis [HAHD]), have demonstrated significant efficacy in reducing the levels of β2 M, parathyroid hormone (PTH), and various cytokines [19,20,21,22]. This reduction correlates with notable improvements in clinical symptoms and outcomes, positioning HAHD as a promising therapeutic option for patients with ESRD [23,24,25]. While HA has been extensively studied for its ability to clear middle molecules, its effectiveness in removing PBUTs remains less well characterized. The pHA130 cartridge (Jafron Biomedical Co., Ltd., Zhuhai, China) is an innovative sorbent device featuring microcontrolled positive charges that mimic the binding sites of uraemic toxins on human serum albumin. This design enables competitive binding and effective removal of both middle-molecular-weight uraemic toxins and PBUTs [26]. This engineered surface chemistry is designed to create a higher affinity for anionic PBUTs, such as IS and PCS, compared with endogenous albumin. The removal is facilitated by a multi-faceted mechanism involving not only the hydrophobic and π-π interactions inherent to its polystyrene backbone but also, crucially, targeted electrostatic attractions and hydrogen bonding between the sorbent’s functional groups and the negatively charged toxins [27]. This combination of interactions enables the competitive dissociation of PBUTs from albumin and their subsequent capture, a mechanism that distinguishes it from conventional neutral macroporous sorbents that rely primarily on less specific hydrophobic interactions.

In light of these issues, the current study was designed as a single-centre, randomized controlled trial to evaluate the efficacy and safety of HAHD using a pHA130 cartridge for the removal of PBUTs and middle-molecular-weight uraemic toxins in patients with ESRD. This investigation aims to generate robust evidence to guide the optimal application of HA in routine clinical practice for the management of uraemic toxicity.

## 2. Results

### 2.1. Patient Characteristics

**Figure 1:**
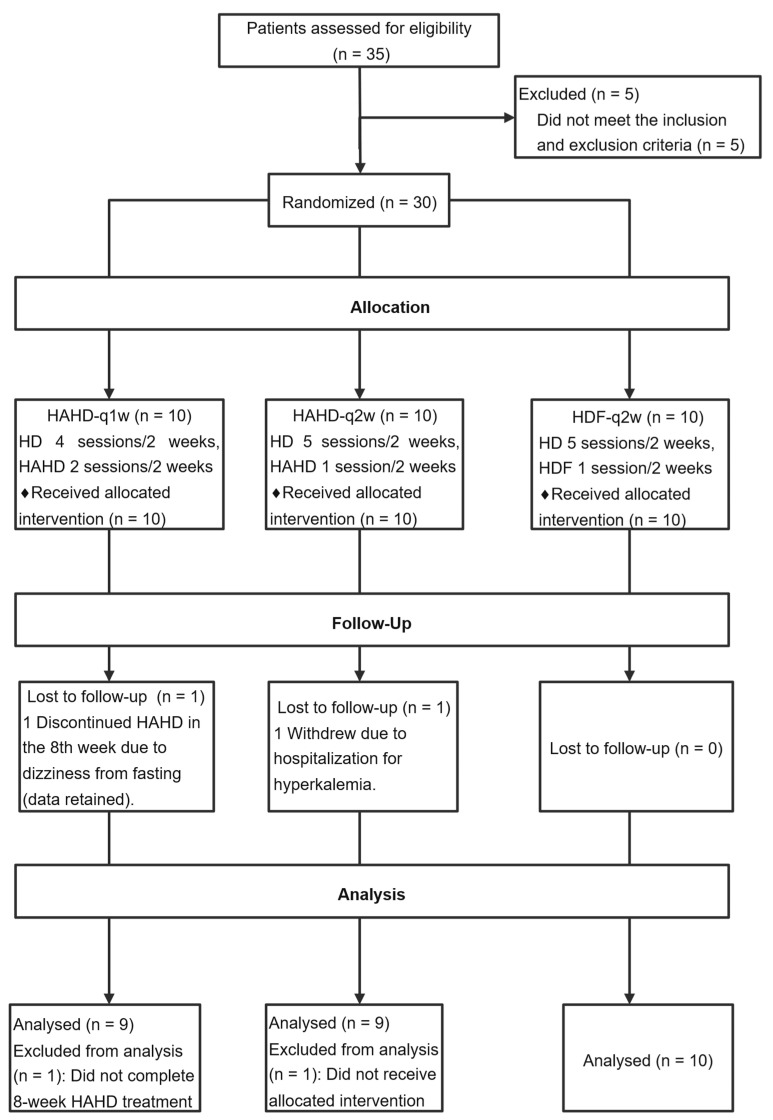
Patient enrolment and follow-up.

A total of 30 patients were randomized equally to the HAHD-q1w (n = 10), HAHD-q2w (n = 10), and HDF (n = 10) groups. Baseline demographics—including age, sex distribution, dialysis vintage, predialysis blood pressure—and key laboratory parameters were well balanced across all three arms (all *p* > 0.05; Table 1). No participants withdrew consent or were lost to follow-up; however, two patients (one in HAHD-q1w and one in HAHD-q2w) were unable to complete the Week 8 RR measurement due to adverse events (see Safety Outcomes); all other safety and secondary endpoint data were successfully collected (Figure 1).

### 2.2. Primary Outcome: Single-Session Toxin Reduction

Compared with the HDF-q2w group (n = 10), the combined HAHD group (n = 20) presented a significantly greater mean RR of IS (46.89 ± 15.54% vs. 31.78 ± 40.07%, *p* = 0.044). Similar superiority for the combined HAHD group was observed for the PCS RR (44.59 ± 10.25% vs. 31.36 ± 21.80% for HDF, *p* = 0.003). No significant difference was observed in the YKL-40 RR (HAHD: 29.83 ± 10.19% vs. HDF: 23.86 ± 26.13%, *p* = 0.336) (Table 2).

### 2.3. Longitudinal Toxin Levels and Frequency Effects

After 8 weeks, weekly HAHD (HAHD-q1w) significantly reduced predialysis IS levels from 33.26 ± 15.32 mg/L to 22.54 ± 9.66 mg/L (*p* = 0.001). Biweekly HAHD (HAHD-q2w) also significantly reduced predialysis IS from 31.15 ± 14.82 mg/L to 21.27 ± 9.85 mg/L (*p* = 0.004). The HDF-q2w group showed a reduction from 44.63 ± 22.78 mg/L to 32.55 ± 19.04 mg/L (*p* = 0.001).

HAHD-q1w demonstrated a sustained, although not statistically significant, reduction in PCS levels (*p* = 0.905 vs. baseline), whereas HAHD-q2w showed a paradoxical increase in PCS (*p* = 0.042). YKL-40 levels remained stable across all groups (Table 3).

### 2.4. Postintervention Rebound

During the 4-week follow-up, the IS levels did not significantly rebound in the HAHD-q1w group (*p* = 0.062), and they remained stable in the HAHD-q2w (*p* = 0.961) and HDF (*p* = 0.803) groups. PCS levels marginally decreased in the HAHD-q1w (*p* = 0.097) and HAHD-q2w (*p* = 0.055) groups but declined significantly in the HDF-q2w group (*p* = 0.045). YKL-40 levels increased significantly in the HDF group (*p* = 0.023) but remained stable in the HAHD group (Table 4).

### 2.5. Patient-Reported Outcomes

HAHD therapy significantly improved pruritus severity (Duo score) and sleep quality (PSQI) (Table 3). At 8 weeks, the HAHD-q1w group presented a 4.70-point mean reduction in Duo score (from 13.00 ± 5.73 to 8.30 ± 3.43, *p* < 0.001) and a 4.50-point mean improvement in PSQI score (from 10.30 ± 3.50 to 5.80 ± 1.87, *p* < 0.001). The HAHD-q2w group demonstrated a 2.33-point mean reduction in Duo score (*p* = 0.009) and a 4.00-point mean improvement in PSQI (*p* < 0.001). In contrast, HDF showed minimal, nonsignificant changes in the Duo score and PSQI (*p* = 0.132 and *p* = 0.724, respectively). These benefits persisted in the HAHD groups at the 4-week follow-up (Table 4).

### 2.6. Generalized Estimating Equation (GEE) Analysis

Longitudinal analysis using GEE was performed to assess changes over the 12-week study period. The models revealed no statistically significant differences between the treatment groups over time for longitudinal predialysis levels of IS, PCS, and YKL-40, or for patient-reported outcome scores (Duo and PSQI) (Table 5).

### 2.7. Safety Outcomes

Adverse events were generally mild and comparable across groups (*p* > 0.05 for overall incidence). One patient in the HAHD-q2w group discontinued treatment at Week 7 because of hospitalization for hyperkalaemia (considered unlikely related to study intervention by the investigator). One patient in the HAHD-q1w group experienced dizziness during one HAHD session (attributed to fasting), resulting in incomplete data collection for that session; however, they completed the study. No hypersensitivity reactions to the pHA130 cartridge were reported.

## 3. Discussion

The current randomized controlled trial demonstrated that HAHD incorporating the pHA130 cartridge achieves superior single-session removal of the key PBUTs, ISs, and PCSs compared with high-volume HDF in patients with ESRD. This finding is clinically relevant, as PBUTs are implicated in the pathogenesis of numerous uraemic complications, including cardiovascular disease and the progression of CKD [1,4]. While HDF is recognized for its enhanced clearance of middle molecules over conventional HD [13], its efficacy for PBUTs is often limited [15]. Our study revealed that pHA130 HAHD offers a significant advantage in this regard, with IS and PCS reduction ratios (46.9% and 44.6%, respectively) surpassing those of HDF (31.8% and 31.4%). However, it is important to interpret this finding with caution. While single-session efficacy was superior, the longitudinal analysis over 8 weeks showed that the reduction in pre-dialysis toxin levels was not significantly different between the HAHD and HDF groups, suggesting that HDF may achieve comparable long-term control, albeit through a different kinetic profile. This enhanced clearance is likely attributable to the specific adsorptive properties of the pHA130 resin, which is designed to have affinity for such toxins, potentially through mechanisms such as controlled positive charges mimicking albumin binding sites [27]. Studies on similar hypercrosslinked polystyrene adsorbents have highlighted their large surface area and hierarchical pore structure, facilitating the capture of a broad range of uraemic toxins. Our clinical data support its in vivo efficacy for PBUTs. This finding is consistent with other studies indicating that certain HA cartridges can effectively remove PBUTs and other toxins that are not well cleared by standard dialysis. For example, Ramírez-Guerrero et al. (2025) reported that HA130 combined with HF-HD significantly increased the removal of carboxymethyllysine (CML), an advanced glycation end product (AGE), compared with HF-HD alone [28].

In addition to toxin removal, our study highlights the patient-centred benefits of HAHD. Weekly HAHD (HAHD-q1w) led to substantial improvements in uraemic pruritus and sleep quality, symptoms that severely impact patients’ quality of life. The magnitude of improvement (Duo score reduction of 36.2%, PSQI improvement of 43.7%) was clinically significant and surpassed that seen with biweekly HAHD or HDF in our trial. This symptomatic relief may be mediated by the enhanced removal of PBUTs such as the IS and PCS, which are known to contribute to pruritus and sleep disturbances, as well as potentially other inflammatory mediators [29]. Several studies have linked HAHD to improvements in pruritus and other uraemic symptoms, which often correlate with reductions in inflammatory markers or specific toxins. The “inflammation mitigation hypothesis” proposed by Ronco et al. suggests that effective removal of uraemic toxins and chemical mediators by HAHD can lead to a reduction in systemic inflammation and a subsequent decrease in the generation of certain toxins, such as β2 M [30]. While we did not measure a broad panel of inflammatory cytokines, the symptomatic improvement observed could be an indirect reflection of such an effect. The observation that HAHD-q1w was more effective than HAHD-q2w in symptom relief underscores the potential importance of treatment frequency for managing these chronic uraemic burdens.

A notable and unexpected finding was the paradoxical increase in predialysis PCS levels in the HAHD-q2w group (*p* = 0.042) at Week 8. This phenomenon was not observed for IS and warrants careful consideration. One possible explanation relates to the complex interplay between toxin generation, compartmental kinetics, and treatment frequency. It is conceivable that the biweekly HAHD regimen, while effective in the short term, is insufficient to counteract the interdialytic rebound and production of PCS, which may have different metabolic pathways and generation rates than IS [31,32]. The transient reduction might trigger a compensatory increase in gut microbial production or release from tissue stores. The differential behaviour between IS and PCS could also reflect varying affinities of the pHA130 sorbent for these two toxins, although in vitro data suggest similar high adsorption rates. This observation underscores the need for further research into the dose-response relationship of HAHD and the distinct kinetics of individual PBUTs to optimize treatment frequency.

The differential strengths of HAHD and HDF were delineated in this study. This reinforces the concept of “personalized dialysis,” where treatment modalities might be chosen or combined on the basis of the specific uraemic toxin profile and clinical phenotype of the patient [33]. For patients predominantly suffering from PBUT-related complications or severe pruritus, HAHD, particularly at a relatively high frequency, might be a preferred option. Practically, this could mean implementing a symptom-based approach, where patients with persistent, severe pruritus (e.g., Duo score > 10) despite optimized standard care could be considered for a trial of weekly HAHD. Alternatively, a biomarker-guided strategy could be envisioned, where patients with markedly elevated predialysis IS or PCS levels could be candidates for this intensified therapy. However, the routine implementation of such a biomarker-guided approach is currently limited by the fact that IS and PCS are not standardly measured in clinical practice. Our findings may therefore help advocate for the inclusion of PBUT monitoring in future clinical guidelines and insurance reimbursement policies, which would be a critical step toward true personalized care. The sustained reduction in pre-dialysis IS levels with both HAHD regimens at Week 8 suggests a meaningful impact on the overall toxin burden, although the GEE analysis did not reveal significant long-term differences in pre-dialysis toxin levels between groups over the entire study period, possibly due to sample size or study duration.

Safety and tolerability are paramount for any new therapeutic strategy. In our study, HAHD with the pHA130 cartridge was well tolerated, with an overall adverse event profile comparable to that of HDF. Importantly, the standard LMWH anticoagulation dose of 60 IU/kg used for HAHD sessions did not lead to increased clotting events compared with HDF. This is a significant practical advantage, as some HA procedures might require adjusted anticoagulation protocols. Recent studies on extended HA durations (e.g., 4-h sessions) have also reported noninferior safety regarding clotting when appropriate anticoagulation and flow rates are maintained [34]. The isolated case of hyperkalaemia leading to discontinuation in the HAHD-q2w group was deemed unrelated to the intervention, and the dizziness episode in the HAHD-q1w group was attributed to fasting. Although deemed unrelated, the occurrence of severe hyperkalemia highlights the importance of vigilant electrolyte monitoring in all patients with ESRD, including those undergoing novel therapies such as HAHD, especially if they have a predisposition to such complications. No hypersensitivity reactions to the pHA130 cartridge occurred. These findings are consistent with reports on newer generation sorbents that exhibit good biocompatibility [24].

Our study has several limitations. The single-centre design and small sample size (n = 30) may limit the generalizability and precision of the effect estimates. Future multicentre trials with larger cohorts are needed. The open-label design, although necessitated by the distinct nature of the interventions, could introduce bias, particularly for patient-reported outcomes; however, laboratory assays were performed by blinded personnel, and statistical analysis was initially conducted by a blinded team. Furthermore, for the primary endpoint analysis, we pooled the two HAHD groups. While this was a prespecified approach to test the efficacy of the device itself, pooling different frequency regimens can be controversial and may mask subtle differences between them. The 8-week intervention and 4-week follow-up are relatively short for assessing long-term clinical impacts such as cardiovascular events or mortality; additionally detailed results for dialysis adequacy (Kt/V, URR) were not presented in this report, which limits a full comparison of small solute clearance adequacy between the groups, although baseline comparability was established. The lack of significant group differences in longitudinal toxin levels in the GEE models, despite significant single-session removal and changes from baseline, warrants further investigation in studies with longer durations and potentially more frequent longitudinal measurements to better understand interdialytic toxin kinetics. Furthermore, the discrepancy between the significant single-session removal of toxins and the lack of significant longitudinal differences between groups in the GEE analysis is a key limitation. This may be due to the short study duration or insufficient frequency of measurements, which may have failed to capture the true chronic effect on pre-dialysis toxin levels. Cost-effectiveness, another important consideration, was not assessed.

## 4. Conclusions

This randomized controlled trial provides preliminary evidence that HAHD with a pHA130 cartridge is superior to HDF for the single-session removal of IS (46.9% vs. 31.8%) and PCS (44.6% vs. 31.4%) and offers significant benefits in alleviating uraemic pruritus and sleep disturbances, particularly with weekly application. The distinct advantages of pHA130 HAHD in managing PBUTs and related symptoms suggest that it is a valuable addition to the spectrum of renal replacement therapies. These findings encourage a personalized approach to dialysis, tailoring the modality to the patient’s specific uraemic toxin profile and symptomatic burden. Further large-scale, long-term studies, ideally with blinded assessment of subjective outcomes, are essential to confirm these benefits for hard clinical outcomes and to establish the optimal role and cost-effectiveness of pHA130 HAHD in the management of patients with ESRD.

## 5. Materials and Methods

### 5.1. Study Design

This single-centre, open-label, randomized controlled trial was conducted at West China Hospital, Sichuan University, between August and November 2024. Eligible participants were patients with ESRD, aged 18–75 years, who were undergoing maintenance HD for at least three months with a thrice-weekly regimen and utilized autologous arteriovenous fistulas or grafts with a blood flow rate (Qb) of ≥200 mL/min. Patients were excluded if they were undergoing concurrent peritoneal dialysis; had recently received HA therapy exceeding one month within the past three months; or had severe cardiovascular, pulmonary, or cerebrovascular diseases; active malignancies; severe bleeding disorders; platelet (PLT) counts < 60 × 10^9^/L; a life expectancy under two years; ongoing participation in other clinical trials; or hypersensitivity to dialysis materials. Written informed consent was obtained from all participants prior to enrolment. The study was conducted in accordance with the Good Clinical Practice guidelines and the principles of the Declaration of Helsinki. The study was approved by the Ethics Committee of West China Hospital (IRB No. 2024-734) and registered with the Chinese Clinical Trial Registry (ChiCTR).

Eligible patients who provided written informed consent were randomly assigned in a 1:1:1 ratio to one of three treatment arms: the HDF-q2w group, HAHD-q2w group, or HAHD-q1w group. The randomization sequence was generated by an independent statistician not involved in patient recruitment or assessment, using a computer-generated random number list. Allocation concealment was maintained using sequentially numbered, opaque, sealed envelopes. These envelopes were prepared by a designated research staff member not involved in patient enrolment and were opened by the enrolling investigator only after a participant had met all eligibility criteria and consented to participate, immediately prior to the first intervention session. This ensured that the treatment allocation was concealed from both the participant and the enrolling investigator until the point of assignment. Given the significant differences in the technical requirements between HA and conventional HD and HDF, the trial was conducted in an open-label manner, meaning that neither the patients nor the treating clinicians were blinded to the group assignments. This design may introduce performance and detection bias, particularly for subjective patient-reported outcomes. To minimize potential biases, all posttreatment blood samples were collected in batches and measured by laboratory staff who were unaware of the intervention groups. Additionally, an independent data-management team, which was also unaware of the treatment assignments, conducted all the statistical analyses of the coded datasets.

### 5.2. Interventions

Following a 1-week screening period, participants were assigned to their allocated intervention for an 8-week period: (i) the HDF-q2w group, receiving standard HD with one session replaced by online predilution HDF every two weeks; (ii) the HAHD-q2w group, in which the standard HD regimen was combined with a pHA130 haemoadsorption session once every two weeks; and (iii) the HAHD-q1w group, in which HD was supplemented by pHA130 haemoadsorption once weekly. Upon completion of the intervention, all participants resumed their standard HD regimen throughout a four-week follow-up period.

### 5.3. Treatment Protocols

HFHD was performed using OCI-HD150 polysulfone dialyzers (surface area, 1.5 m^2^; ultrafiltration coefficient, 48 mL/h/mmHg), with the blood flow rate (Qb) maintained at 200–300 mL/min. The dialysate flow rate was set at 500 mL/min, and anticoagulation was achieved with low-molecular-weight heparin (LMWH). Each session lasted 4 h, with a maximum ultrafiltration volume not exceeding 5% of body weight.

HDF was conducted using Revaclear 300 dialyzers with an online-produced replacement fluid volume of 15–25 L. Qb was maintained at 200–300 mL/min, with a dialysate flow rate of 700 mL/min. Predilution substitution was performed at a rate adjusted according to the blood flow speed.

During HAHD treatment sessions, the pHA130 HA cartridge was incorporated into the extracorporeal circuit before the dialyzer. HA was conducted for the initial 2 h, followed by standard HD for the remaining 2 h. Qb during HA was set at 200–250 mL/min, increasing to 200–300 mL/min for the post-HA phase. Anticoagulation was performed with LMWH at a dose of 60 IU/kg, and standard dialysate composition was maintained throughout.

### 5.4. Outcomes

The primary endpoint of this study was the single-session reduction ratio (RR) of total serum IS, typically assessed from representative sessions at Week 4 and Week 8 of the intervention period. RR was calculated using the formula RR (%) = (1 − (cCpost/Cpre)) × 100, where Cpre and Cpost are the pre- and posttreatment concentrations of the IS, respectively. Cpost values were corrected for the degree of haemoconcentration and the approximate extracellular fluid volume using the formula derived by Bergström and Wehle [35]: corrected postdialysis concentration = measured postdialysis concentration/[1 + ((predialysis weight − postdialysis weight)/(0.2 × dry weight))].

The secondary endpoints included (i) the single-session RRs of total serum PCS and YKL-40, which were evaluated through similar reduction ratio calculations from samples taken at Week 4 and Week 8; (ii) longitudinal changes in predialysis serum concentrations of IS, PCS, and YKL-40, which were assessed at baseline, Week 4, Week 8, and at the end of the 4-week follow-up (Week 12); (iii) changes from baseline in patient-reported outcomes: Duo pruritus score (points) and PSQI score (points), which were assessed at baseline, Week 4, Week 8, and Week 12; and (iv) safety and tolerability, which were assessed by monitoring the incidence and nature of adverse events (AEs) throughout the study, including intradialytic hypotension, hypersensitivity reactions, and clotting events within the extracorporeal circuit.

### 5.5. Statistical Analysis

Based on previous literature and preliminary data, the RR of IS following HDF was anticipated to be 24.2% (SD = 10.7%), whereas the IS RR in patients treated with pHA130 HAHD was expected to reach 45.51% (SD = 13.75%). Using a two-sided significance level of α = 0.05 and a statistical power (1-β) of 80%, the minimum required sample size was calculated to be approximately 7 patients per group for a two-group comparison. To account for potential dropouts and to enable three-group comparisons, the sample size was increased to 10 patients per group, resulting in a total enrolment of 30 patients.

For the analysis of the primary endpoint, the HAHD-q2w and HAHD-q1w groups were pooled into a combined HAHD group (n = 20) for comparison against the HDF-q2w group (n = 10). This approach was prespecified to increase the statistical power for detecting a difference in single-session efficacy between the novel HAHD therapy (utilizing the pHA130 cartridge) and standard HDF for this primary measure of immediate toxin removal. The rationale was that the intrinsic adsorptive performance of the pHA130 cartridge within a single session was hypothesized to be the principal determinant of the single-session RR, whereas the frequency of HAHD application (weekly vs. biweekly) was anticipated to primarily influence longer-term, cumulative effects and predialysis toxin concentrations, which were assessed as key secondary outcomes.

Continuous variables conforming to a normal distribution are presented as means ± SDs and were compared using independent-samples t tests (two groups) or one-way analysis of variance (ANOVA) (three groups). Nonnormally distributed data are expressed as medians with interquartile ranges (IQRs) and were analysed using the Mann–Whitney U test (two groups) or the Kruskal-Wallis test (three groups). Categorical variables are summarized as frequencies and percentages and were compared using the chi-square test or Fisher’s exact test. As multiple secondary endpoints (PCS, YKL-40) were tested, there is a potential for an inflated Type I error rate. The results of these secondary analyses should be considered exploratory and hypothesis-generating rather than confirmatory.

To further investigate associations between the treatment group and longitudinal outcomes, generalized estimating equation (GEE) models were used, accounting for repeated measures at baseline, Week 4, Week 8, and Week 12. GEE models are an extension of generalized linear models used for analysing longitudinal or clustered data. They are particularly useful when the outcome variable is not normally distributed and when observations within a subject are correlated over time. By specifying a correlation structure, the GEE model provides valid standard errors for the regression coefficients, even if the correlation structure is misspecified. A first-order autoregressive (AR1, which assumes that correlations between observations decrease as the time between them increases) correlation matrix was adopted. The linear regression coefficient B and its 95% CI are reported. All analyses were performed on an intention-to-treat (ITT) basis using R software (version 4.4.0). *p* value < 0.05 (two-tailed) was considered statistically significant. Missing data were handled using available case analysis for endpoint comparisons; GEE models inherently account for missing data under the assumption of missing data at random.

## Figures and Tables

**Table 1:** Baseline demographic, clinical, and laboratory characteristics of patients by treatment group. The data are presented as the means ± standard deviations or n (%). Comparisons across groups (weekly haemoadsorption (HAHD-q1w), biweekly haemoadsorption (HAHD-q2w), and biweekly haemodiafiltration (HDF)) were performed using ANOVA or Kruskal-Wallis tests for continuous variables and chi-square test for categorical variables.

Parameters	HAHD-q1w (n = 10)	HAHD-q2w (n = 10)	HDF-q2w (n = 10)	All (n = 30)	F	*p*
Age (years)	52.50 ± 8.82	52.30 ± 11.25	51.60 ± 14.03	52.13 ± 11.16	0.02	0.983
Dialysis duration (months)	78.10 ± 42.81	82.40 ± 38.20	66.40 ± 42.17	75.63 ± 40.26	0.41	0.671
Systolic blood pressure (SBP) (mmHg)	142.40 ± 10.86	135.70 ± 12.37	137 ± 15.51	138.37 ± 12.94	0.74	0.486
Diastolic blood pressure (DBP) (mmHg)	78.90 ± 10.57	73.80 ± 11.42	78.60 ± 12.88	77.10 ± 11.50	0.60	0.555
Pre-dialysis heart rate (beats/min)	77.80 ± 13.81	77.40 ± 10.94	73.30 ± 7.30	76.17 ± 10.82	0.51	0.605
Red blood cell (RBC) count (×10^12^/L)	3.53 ± 0.70	3.76 ± 0.80	3.76 ± 0.56	3.68 ± 0.68	0.38	0.689
Packed cell volume (PCV) (L/L)	0.34 ± 0.06	0.35 ± 0.03	0.36 ± 0.04	0.35 ± 0.05	0.39	0.679
White blood cell (WBC) count (×10^9^/L)	6.05 ± 1.27	5.77 ± 1.14	5.89 ± 0.74	5.90 ± 1.04	0.18	0.837
Hemoglobin (Hb) (g/L)	106.50 ± 20.10	109.50 ± 7.11	114.50 ± 13.45	110.17 ± 14.44	0.77	0.472
Platelet (PLT) count (×10^9^/L)	146.30 ± 47.75	161.40 ± 29.36	171.20 ± 32.39	159.63 ± 37.54	1.13	0.339
Previous heparin dosage (mL)	0.33 ± 0.05	0.34 ± 0.04	0.31 ± 0.07	0.32 ± 0.05	0.40	0.677
Baseline heparin dosage (mL)	0.37 ± 0.05	0.34 ± 0.04	0.32 ± 0.07	0.34 ± 0.06	2.61	0.092
Pre-dialysis body weight (kg)	65.55 ± 8.24	64.93 ± 10.85	61.59 ± 8.19	64.02 ± 9.03	0.54	0.590
Post-dialysis body weight (kg)	63.46 ± 7.65	62.66 ± 10.93	59.47 ± 7.92	61.86 ± 8.82	0.56	0.580
Interdialytic weight gain (kg)	2.09 ± 1.05	2.27 ± 0.73	2.12 ± 0.78	2.16 ± 0.84	0.12	0.884
Duo Pruritus Score (points)	13 ± 5.73	8.80 ± 2.62	9.50 ± 5.04	10.43 ± 4.87	2.33	0.116
PSQI score (points)	10.30 ± 3.50	9.60 ± 2.67	7.50 ± 3.60	9.13 ± 3.39	1.97	0.159
Pre-dialysis Indoxyl Sulfate (mg/L)	33.26 ± 15.32	31.26 ± 13.97	44.63 ± 22.78	36.38 ± 18.18	1.64	0.212
Pre-dialysis p-Cresyl Sulfate (mg/L)	17.79 ± 23.70	16.70 ± 13.29	30.88 ± 22.53	21.79 ± 20.73	1.50	0.241
Pre-dialysis β2-Microglobulin (mg/L)	32.69 ± 4.54	31.55 ± 3.25	33.12 ± 3.51	32.45 ± 3.74	0.45	0.640
Pre-dialysis YKL-40 (pg/mL)	260.53 ± 162.05	253.55 ± 173.99	242.20 ± 86.07	252.09 ± 141.08	0.04	0.961

**Table 2:** Comparison of the single-session reduction ratios of uraemic toxins between the haemoadsorption (HAHD) and haemodiafiltration (HDF) groups. Reduction ratios (%) for indoxyl sulphate (IS), para-cresyl sulphate (PCS), YKL-40, and the combined HAHD group (n = 38 sessions) versus the HDF group (n = 20 sessions). Assessments were typically based on Week 4 and Week 8 sessions. The data are presented as the means ± standard deviations. Statistical comparisons were performed using independent samples *t*-tests.

Parameters	HAHD Group (n = 38)	HDF Group (n = 20)	All (n = 58)	t	*p*
IS RR (%)	46.89 ± 15.54	31.78 ± 40.07	41.68 ± 27.28	2.06	0.044
PCS RR (%)	44.59 ± 10.25	31.36 ± 21.80	40.03 ± 16.34	3.15	0.003
YKL-40 RR (%)	29.83 ± 10.19	23.86 ± 26.13	27.77 ± 17.41	0.98	0.336

**Table 3:** Longitudinal changes in predialysis laboratory parameters after 8 weeks of treatment across groups. Baseline and 8-week post-treatment values for red blood cell count (RBC), packed cell volume (PCV), white blood cell count (WBC), haemoglobin (Hb), platelet count (PLT), pruritus severity (Duo score), sleep quality (Pittsburgh Sleep Quality Index (PSQI)), and uraemic toxin levels (IS, PCS, YKL-40) in weekly HAHD (HAHD-q1w), biweekly HAHD (HAHD-q2w), and HDF groups. The data are presented as the means ± standard deviations. Within-group comparisons were performed using paired t-tests; between-group comparisons were performed using ANOVA.

Parameters	HAHD-q1w (n = 10)			HAHD-q2w (n = 10)			HDF-q2w (n = 10)		
	Baseline	After 8 Weeks	*p*	Baseline	After 8 Weeks	*p*	Baseline	After 8 Weeks	*p*
Red blood cell count (×10^12^/L)	3.53 ± 0.70	3.42 ± 0.68	0.709	3.84 ± 0.81	3.95 ± 0.88	0.805	3.76 ± 0.56	3.79 ± 0.38	0.873
Packed cell volume (L/L)	0.34 ± 0.06	0.33 ± 0.06	0.589	0.35 ± 0.03	0.36 ± 0.04	0.592	0.36 ± 0.04	0.37 ± 0.02	0.753
White blood cell count (×10^9^/L)	6.05 ± 1.27	6.99 ± 1.46	0.094	5.68 ± 1.17	6.87 ± 1.58	0.154	5.89 ± 0.74	6.37 ± 0.84	0.202
Hemoglobin (g/L)	106.50 ± 20.10	102.70 ± 18.43	0.559	110.33 ± 7.00	112.44 ± 9.61	0.623	114.50 ± 13.45	116.10 ± 8.03	0.759
Platelet count (×10^9^/L)	146.30 ± 47.75	156.60 ± 43.74	0.444	162.44 ± 30.95	164.89 ± 34.41	0.854	171.20 ± 32.39	182.10 ± 34.80	0.485
Duo pruritus score (points)	13.00 ± 5.73	8.30 ± 3.43	<0.001	8.44 ± 2.51	6.11 ± 0.93	0.009	9.50 ± 5.04	8.80 ± 5.71	0.132
PSQI (points)	10.30 ± 3.50	5.80 ± 1.87	<0.001	9.67 ± 2.83	5.67 ± 1.66	<0.001	7.50 ± 3.60	7.90 ± 4.68	0.724
Indoxyl sulfate (mg/L)	33.26 ± 15.32	22.54 ± 9.66	0.001	31.15 ± 14.82	21.27 ± 9.85	0.004	44.63 ± 22.78	32.55 ± 19.04	0.001
p-Cresyl sulfate (mg/L)	17.79 ± 23.70	17.41 ± 17.47	0.905	14.86 ± 12.68	20.22 ± 14.53	0.042	30.88 ± 22.53	29.32 ± 19.66	0.736
YKL-40 (pg/mL)	260.53 ± 162.05	284.91 ± 206.00	0.271	253.55 ± 173.99	225.51 ± 131.17	0.318	242.20 ± 86.07	207.54 ± 111.39	0.381

**Table 4:** Postintervention effects on uraemic toxin levels and clinical parameters over 4 weeks following treatment cessation. Changes in laboratory parameters from Week 8 (postintervention) to Week 12 (4-week follow-up) in the weekly HAHD (HAHD-q1w), biweekly HAHD (HAHD-q2w), and HDF groups. The data are presented as the means ± standard deviations. Rebound effects were assessed using paired t tests within groups.

Parameters	HAHD-q1w (n = 10)			HAHD-q2w (n = 10)			HDF-q2w (n = 10)		
	Week 8	Week 12	*p*	Week 8	Week 12	*p*	Week 8	Week 12	*p*
Red blood cell count (×10^12^/L)	3.42 ± 0.68	3.48 ± 0.73	0.410	3.95 ± 0.88	4.09 ± 0.67	0.316	3.79 ± 0.38	3.76 ± 0.36	0.707
Packed cell volume (L/L)	0.33 ± 0.06	0.34 ± 0.07	0.485	0.36 ± 0.04	0.37 ± 0.03	0.211	0.37 ± 0.02	0.36 ± 0.02	0.780
White blood cell count (×10^9^/L)	6.99 ± 1.46	6.08 ± 1.67	0.011	6.87 ± 1.58	6.86 ± 1.84	0.979	6.37 ± 0.84	5.97 ± 1.08	0.239
Hemoglobin (g/L)	102.70 ± 18.43	104.50 ± 21.54	0.473	112.44 ± 9.61	117.44 ± 12.75	0.228	116.10 ± 8.03	114.80 ± 6.44	0.614
Platelet count (×10^9^/L)	156.60 ± 43.74	160.60 ± 45.27	0.549	164.89 ± 34.41	171.67 ± 34.76	0.208	182.10 ± 34.80	179.30 ± 40.65	0.737
Duo pruritus score (points)	8.30 ± 3.43	5.40 ± 3.34	<0.001	6.11 ± 0.93	4.22 ± 1.86	0.003	8.80 ± 5.71	10.00 ± 4.57	0.154
PSQI (points)	5.80 ± 1.87	4.50 ± 2.46	0.057	5.67 ± 1.66	4.44 ± 2.13	0.038	7.90 ± 4.68	8.60 ± 6.38	0.322
Indoxyl sulfate (mg/L)	22.54 ± 9.66	26.34 ± 11.32	0.062	21.27 ± 9.85	21.14 ± 12.45	0.961	32.55 ± 19.04	31.70 ± 14.44	0.803
p-Cresyl sulfate (mg/L)	17.41 ± 17.47	14.47 ± 18.23	0.097	20.22 ± 14.53	14.46 ± 12.32	0.055	29.32 ± 19.66	21.10 ± 14.05	0.045
YKL-40 (pg/mL)	284.91 ± 206.00	260.36 ± 177.70	0.185	225.51 ± 131.17	256.96 ± 143.16	0.190	207.54 ± 111.39	243.90 ± 97.00	0.023

**Table 5:** Generalized estimating equation (GEE) analysis of vital signs, laboratory parameters, and clinical outcomes across treatment groups. Estimated regression coefficients (B) and 95% confidence intervals (CIs) for longitudinal comparisons of Duo scores and PSQI, IS, PCS, and YKL-40 scores between groups. Analyses were adjusted for repeated measures using an autoregressive correlation matrix (AR1).

	Group	Estimation (B)	S.E.	95% CI	*p*
Duo pruritus score (points)	HDF	0	-	-	-
	HAHD-q1w	1.53	2.10	−2.58 to 5.64	0.465
	HAHD-q2w	−1.44	1.71	−4.80 to 1.92	0.401
PSQI (points)	HDF	0	-	-	-
	HAHD-q1w	0.44	1.39	−2.30 to 3.17	0.754
	HAHD-q2w	0.05	1.30	−2.49 to 2.60	0.967
Indoxyl sulfate (mg/L)	HDF	0	-	-	-
	HAHD-q1w	−10.63	7.12	−24.59 to 3.32	0.135
	HAHD-q2w	−11.26	7.03	−25.04 to 2.52	0.109
p-Cresyl sulfate (mg/L)	HDF	0	-	-	-
	HAHD-q1w	−12.03	8.17	−28.05 to 3.99	0.141
	HAHD-q2w	−10.77	6.82	−24.13 to 2.60	0.114
YKL-40 (pg/mL)	HDF	0	-	-	-
	HAHD-q1w	49.09	59.90	−68.32 to 166.50	0.413
	HAHD-q2w	15.93	49.93	−81.93 to 113.78	0.750

## Data Availability

The data presented in this study are available on request from the corresponding author. The data are not publicly available due to privacy and ethical restrictions.
